# Antibody trapping: A novel mechanism of parasite immune evasion by the trematode *Echinostoma caproni*

**DOI:** 10.1371/journal.pntd.0005773

**Published:** 2017-07-17

**Authors:** Alba Cortés, Javier Sotillo, Carla Muñoz-Antolí, Javier Molina-Durán, J. Guillermo Esteban, Rafael Toledo

**Affiliations:** 1 Departamento de Parasitología, Facultad de Farmacia, Universidad de Valencia, Burjassot, Valencia, Spain; 2 Centre for Biodiscovery and Molecular Development of Therapeutics, Australian Institute of Tropical Health and Medicine, James Cook University, Cairns, Queensland, Australia; University of Edinburgh, UNITED KINGDOM

## Abstract

**Background:**

Helminth infections are among the most prevalent neglected tropical diseases, causing an enormous impact in global health and the socioeconomic growth of developing countries. In this context, the study of helminth biology, with emphasis on host-parasite interactions, appears as a promising approach for developing new tools to prevent and control these infections.

**Methods/Principal findings:**

The role that antibody responses have on helminth infections is still not well understood. To go in depth into this issue, work on the intestinal helminth *Echinostoma caproni* (Trematoda: Echinostomatidae) has been undertaken. Adult parasites were recovered from infected mice and cultured *in vitro*. Double indirect immunofluorescence at increasing culture times was done to show that *in vivo*-bound surface antibodies become trapped within a layer of excretory/secretory products that covers the parasite. Entrapped antibodies are then degraded by parasite-derived proteases, since protease inhibitors prevent for antibody loss in culture. Electron microscopy and immunogold-labelling of secreted proteins provide evidence that this mechanism is consistent with tegument dynamics and ultrastructure, hence it is feasible to occur *in vivo*. Secretory vesicles discharge their content to the outside and released products are deposited over the parasite surface enabling antibody trapping.

**Conclusion/Significance:**

At the site of infection, both parasite secretion and antibody binding occur simultaneously and constantly. The continuous entrapment of bound antibodies with newly secreted products may serve to minimize the deleterious effects of the antibody-mediated attack. This mechanism of immune evasion may aid to understand the limited effect that antibody responses have in helminth infections, and may contribute to the basis for vaccine development against these highly prevalent diseases.

## Author summary

Helminthiases are highly prevalent neglected tropical diseases, affecting millions of people worldwide, mainly in the poorest regions. The lack of vaccines against these infections is one of the major constraints in the current parasitology and massive efforts are being done in that direction. Herein, we present a potential mechanism for parasite immune evasion consisting in trapping of surface-bound antibodies within the excretory/secretory products that are deposited over the parasite. This mechanism is aided by parasite-derived proteases, well documented virulence factors that degrade the entrapped antibodies. Altogether, this parasite strategy may serve to minimize the antibody-mediated response and promote the development of chronic infections. The present study has been done using the model trematode *Echinostoma caproni*, though is expected to work in other helminths, even in other groups of extracellular pathogens. This opens new expectative to better understanding of host-parasite interactions and susceptibility to helminth infections. Therefore, the results presented in this manuscript may contribute to the basis of anti-helminth vaccine development.

## Introduction

Parasites are able to actively evade or manipulate the host immune system for their own benefit, either increasing their transmission or reducing clearance. That is crucial in the evolution of host-parasite interactions, pathology and virulence. Evasion strategies, such as antigenic variation, antigen masking, molecular mimicry or protease secretion are common among both protozoa and helminth parasites [1]. Ultimately, these mechanisms let the parasites disrupt or manipulate the host immune responses, both innate and adaptive, and/or prevent the formation of a memory response [1]. It is well known that antibodies can affect the development of helminth parasites by hindering processes such as attachment, feeding or motility, among others [2]. Several studies show that antibodies are able to target parasites, such as *Echinostoma caproni* or *Nippostrongylus brasiliensis*, for other immune effector mechanisms such as granulocyte and macrophage binding, or complement system activation [3–5]. Nevertheless, though antibody responses are commonly needed for controlling helminth infections, generally these are not sufficient to prevent nor overcome the infection [2].

The tegument of trematodes is a highly active structure with a key role in host-parasite interactions and the maintenance of tegument integrity is crucial for worm survival [6]. For these reasons, a number of tegumental proteins have been proposed as promising vaccine candidates against these helminthiases. However, though different levels of protection have been observed, antibody responses *per se* normally have limited effect and complete protection against infection has not been reached so far [7–9]. This suggests the existence of intrinsic mechanisms that limit the susceptibility of the tegument to the immune attack. Herein, we describe a potential novel mechanism for parasite immune evasion, which consists in the entrapment of surface-bound antibodies to limit the effects mediated by the humoral response.

*E*. *caproni* is an intestinal trematode, broadly employed as an experimental model for the study of the biology of this group of parasitic helminths, with emphasis on the host-parasite interactions. One of the key features that makes this trematode a suitable model for studying host-parasite relationships is its different compatibility among laboratory rodents [10]. Low-compatible hosts, i.e. rats or jirds, are able to rapidly expel the parasites. Conversely, hosts of high compatibility, such as mice or hamsters, develop chronic infections lasting more than 25 weeks [10–12]. In highly compatible hosts, such as mice, strong, Th1-type inflammatory responses are developed at the site of infection, together with elevated levels of oxidative stress and mucosal antibodies [13,14]. This response, however, is not effective in the clearance of the infection and does not affect worm establishment nor development [10–14]. Worm recovery rates in mice are high, and adults are larger and more fecund than those recovered from hosts of low compatibility are [12]. Altogether, these facts suggest that the parasite is well adapted to this environment and it is capable of avoiding, or minimizing somehow, the deleterious effects mediated by the immune response, including antibodies, developed in mice. Thereupon, the experimental model *E*. *caproni*-mouse have been used herein to further study the mechanism through which parasites are able to withstand the immune response and ensure their survival inside the host.

## Materials and methods

### Experimental infections

The strain of *E*. *caproni* employed and the infection procedures have been described previously [15]. Briefly, encysted metacercariae were removed from kidneys and periacardial cavities of experimentally infected *Biomphalaria glabrata* snails and used for infection. CD1 mice (male, 30–35 g) were infected by gastric gavage with 75 metacercariae of *E*. *caproni*. At 4 weeks post-infection mice were necropsied and the small intestine was longitudinally opened to collect the adult parasites.

### Ethics statement

The animals were maintained under conventional conditions with food and water ad libitum. This study has been approved by the Ethical Committee of Animal Welfare and Experimentation of the University of Valencia (Ref#A18348501775). Protocols adhered to Spanish (Real Decreto 53/2013) and European (2010/63/UE) regulations.

### Double indirect immunofluorescence

*E*. *caproni* adults were fixed by immersion in 4% paraformaldehyde, either immediately after isolation (0 min) or after incubation in RPMI 1640 culture medium (Life Technologies), at 37°C, during increasing time intervals (15, 30, 60 and 120 min). The immunostaining was performed as follows. Briefly, adults were blocked for unspecific unions in 5% BSA (Sigma-Aldrich) in PBS for 1 h, and then incubated for 1h 30 min with a mixture of two primary antibodies, which consisted of: 1) rabbit sera against either *E*. *caproni*-actin [16, 17] or *E*. *caproni*-enolase [16, 18] and 2) goat anti-mouse IgA or goat anti-mouse IgG, both conjugated with HRP (Nordic). Antibody solutions were prepared in PBS by mixing one of the antibodies in 1 (rabbit against *E*. *caproni* protein) and another one from 2 (goat against mouse immunoglobulin), both diluted 1/50 in the final mixture. Different combinations of these antibodies were used to confirm that staining patterns do not depend on specific parasite antigens nor immunoglobulin isotypes, i.e. that different parasite-secreted proteins and/or different antibody isotypes share a common pattern regarding the trapping process.

After carefully washing in PBS (3 times of 10 min each), adults were incubated simultaneously with 2 secondary antibodies: 1) goat anti-rabbit IgG conjugated with Alexa Fluor 647, which tagged rabbit antibodies specifically bound to parasite antigens in the previous step, and 2) goat anti-HRP conjugated with FITC, tagging the HRP-conjugated goat antibodies bound to mouse immunoglobulins. This incubation was performed for one hour in the dark and parasite specimens were washed again in PBS before their examination by confocal microscopy. Secondary antibodies (both from Jackson ImmunoResearch) were diluted to a final concentration of 1/250 each. All incubations were performed at room temperature, under gentle agitation. Antibody solutions were prepared in PBS and no detergents were employed to permeate the samples. Negative controls, employed to set acquisition parameters for confocal microscopy, were performed likewise, excepting the incubation with primary antibodies.

Specific anti-actin and anti-enolase antibodies were prepared in our laboratory through immunization of New Zealand white rabbits with recombinant proteins as described in [18]. Antibody specificity is proved herein by western blot (see below).

Fluorescent staining was visualized by laser scanning confocal microscopy on 10 specimens at each time point. Adult worms were obtained from 3 experimentally infected mice and randomly allocated in the different experimental groups (i.e. times of *in vitro* incubation), so that each group comprised adults from the different hosts. Images were analysed using FV10-ASW 4.2 and Imaris software.

The loss of *in vivo* bound antibodies on worm surface along time was quantitated using ImageJ software to calculate the percentage of image area covered by the fluorescent tag (FITC). Confocal micrographs (x400) were stacked to create Z projections that were converted into binary (black and white) images. Raw integrated density (RawIntDen), which is the sum of the values of all the pixels in the image, was measured and used to calculate the percentage of area covered by the fluorescent tag (% AC) according to the following formula, in which 255 is the density value of a positive (tagged) pixel in the binary image and areas are expressed in pixels:

$$\%\;AC=\;\frac{RawIntDen/255}{Total\;area}\cdot 100$$

Statistical significance in relation to non-incubated worms (0 min) was assessed by unpaired Student’s t test (*p*<0.05). Prior to analysis data normality was confirmed by Shapiro-Wilk test.

To address the role of parasite-derived proteases, adult specimens were incubated for 2 h in RPMI in the presence or absence of a cocktail of protease inhibitors (cOmplete, Mini, EDTA-free, Roche) added at proper concentrations from a 7x stock solution, following the manufacturer’s instructions. The worms were processed as described above and the immunostaining compared between the two groups. To see if *E*. *caproni* adults are susceptible of being recognized by specific antibodies after *in vitro* incubation, the parasites were incubated for 4 h in RPMI to totally eliminate *in vivo*-bound antibodies. After blocking, the worms were incubated for 1 h with the serum of *E*. *caproni*-infected mice, before to proceed with the general protocol for double indirect immunofluorescence using anti-mouse IgG as primary antibody. Pre-immune mouse serum was employed for negative controls.

### Sample preparation for scanning electron microscopy

For SEM, *E*. *caproni* adults were fixed in Karnovsky’s fixative (0.5 M glutaraldehyde, 2.5 M formaldehyde), washed in buffer solution and post-fixed in 2% osmium tetroxide in 0.1 M sodium phosphate buffer, pH 7.2, for 2 h before dehydration by critical point. Mounted specimens were sputter-coated with gold-palladium and examined in a Hitachi S4100 scanning electron microscope at 5 kV.

### Sample preparation for transmission electron microscopy and immunogold staining

Inclusion in LR-white resin for TEM was performed by fixing the adult parasites in glutaraldehyde 2.5% overnight, washing them in phosphate buffer 0.1 M pH 7.2, and then post-fixing in 2% osmium tetroxide in phosphate buffer for 2 h. After several washes in water, parasites were sequentially dehydrated in 30%, 50%, 70% and 96% ethanol, 5 min each. Finally, the worms were sequentially incubated for 2 h in 33% LR-white resin in 96% ethanol, 66% LR-white resin in 96% ethanol, 66% LR-white resin in 100% ethanol and 100% LR-white resin in 100% ethanol. Samples were filtered in resin and polymerized at 60 uC for 48 h. Ultra-thin slices (60 nm) were stained with 2% uranyl acetate prior to visualization by TEM at 70 kV in a microscope Jeol JEM1010. Images were acquired using a digital camera MegaView III with Olympus Image Analysis software.

For the immunogold assay, *E*. *caproni* adults were fixed in Karnovsky’s fixative and included in LR-resin as described above. Grids were washed 5 times, 1 min each, in 20 mM Tris-HCl buffer, pH 8.2, (TB) containing 0.1% BSA and 0.05% Tween-20 and blocked for unspecific unions using goat serum, diluted 1:20 in TB, for 30 min. After washing, free aldehyde groups were blocked for 5 min in TB containing 0.02 M glycine, and washed again. Rabbit sera against *E*. *caproni* actin or *E*. *caproni* excretory/secretory products (ESPs) [17], diluted 1/10 in TB-0.1% BSA, was applied as primary antibody at 4°C overnight. Grids were washed as previously described and incubated for 1 h with gold-labelled secondary antibody, donkey anti-rabbit IgG coupled to 12 nm gold particles (Jackson ImmunoResearch), diluted 1/20 in TB. For double immunogold, samples were processed likewise, using rabbit sera against *E*. *caproni* ESPs as a primary antibody and a mixture of 2 secondary antibodies, which consisted of the same donkey anti-rabbit IgG described above plus a donkey anti-mouse IgG conjugated with 18 nm colloidal gold (Jackson ImmunoResearch), both diluted 1/20 in TB. Negative controls were performed using grids incubated with pre-immune rabbit sera as primary antibody.

### Western-blot confirmation of antibody specificity

The specificity of the primary antibodies employed herein was confirmed by protein electrophoresis in SDS-PAGE and western blot (S1 Fig). A total of 30 μg of ESPs, obtained as previously described [16], were loaded onto 4% stacking and 12% resolving polyacrylamide gels and electrophoresed in Tris-glycine SDS buffer. Proteins were elctrotransferred onto nitrocellulose membranes (0.45 μm) in 20 mM Tris, 192 mM glycine and 20% methanol buffer, pH 8.3, for 90 min at 200 mA. After 1 h blocking in 5% skimmed milk in PBS containing 0.05% of Tween-20 (PBS-T) at room temperature, blots were incubated overnight at 4°C in PBS-T containing each antiserum: rabbit polyclonal anti-*E*. *caproni* actin (1/2,000) [16, 17]; rabbit polyclonal anti-*E*. *caproni* enolase (1/2,000) [16, 18]; and rabbit polyclonal anti-*E*. *caproni* ESPs (1/4,000). Membranes were washed and probed with peroxidase-conjugated secondary antibody, goat anti-rabbit IgG in PBS-T (diluted 1/10,000 for actin and enolase, and 1/20,000 for complete ESPs) for 2 h at room temperature. Negative control was performed likewise, by incubating ESPs against serum of pre-immune rabbit (1/2,000) and secondary antibody (1/10,000). Blots were developed with Amersham ECL Advance Western Blotting Detection Kit (GE Healthcare) following the manufacturer’s instructions and images were taken with ChemiDoc Imaging System (Bio-Rad). The results of this trial are shown in S1 Fig.

## Results and discussion

**Fig 1:**
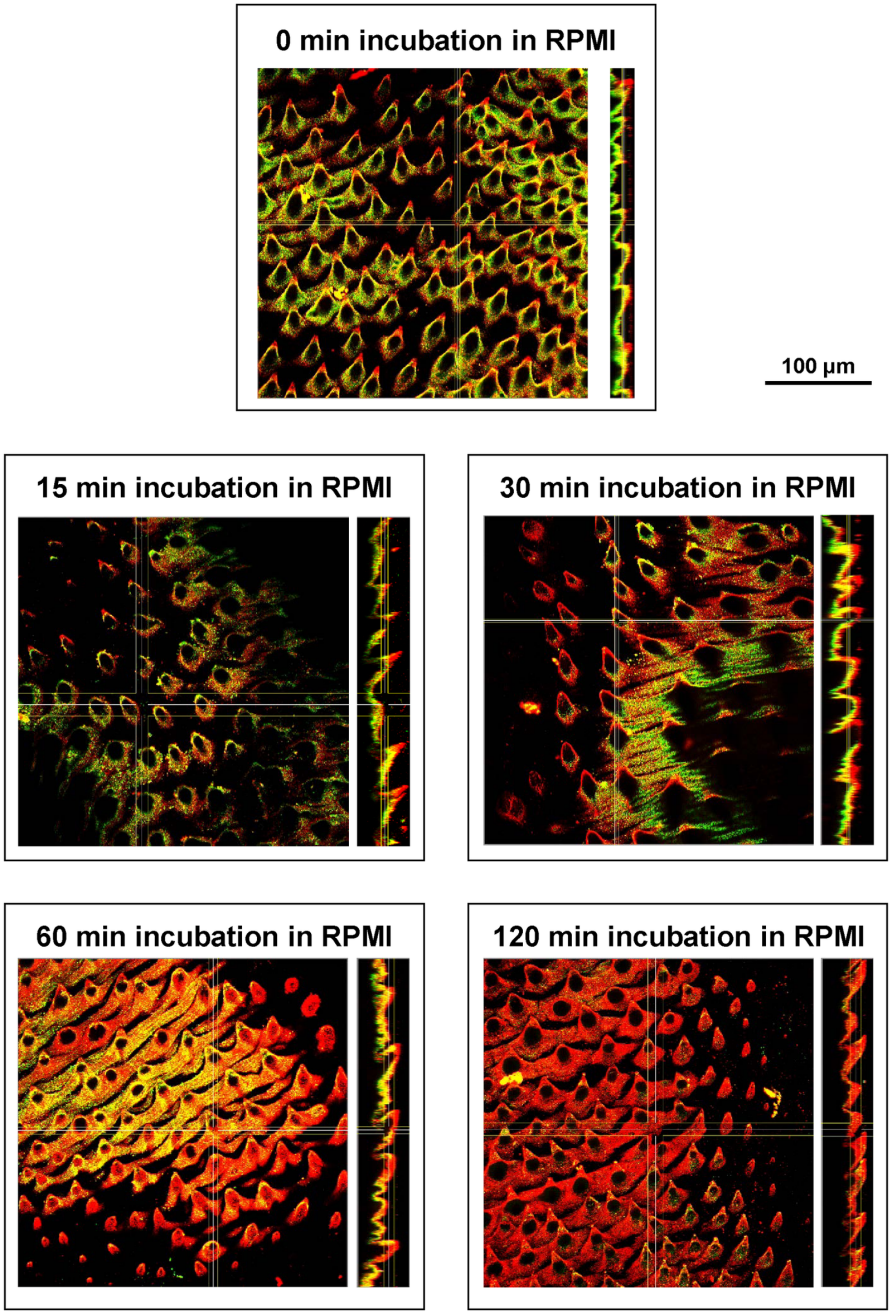
Staining of *Echinostoma caproni* surface at increasing time of *in vitro* incubation. 2D-images from laser confocal microscopy on the surface of *E*. *caproni* adults stained with anti-*E*. *caproni* actin (red) and anti-mouse IgA (green). Merge is shown in yellow. For each time point, representative images of XY and YZ axes are shown (left and right, respectively). Zero minutes incubation refers to worms that were fixed immediately after being removed from the intestine of the mouse.

High levels of mucosal antibodies are characteristic of *E*. *caproni* infections in mice [13]. With the aim to confirm the *in vivo* binding of luminal antibodies over the parasite surface, double indirect immunofluorescence was performed on *E*. *caproni* adults. Specific mouse IgA and IgG were detected on worms at 0 min, indicating that they are susceptible of being affected by antibody-mediated responses (Fig 1, S1 Movie, S2 and S3 Figs). At this time point, an intense staining with the different tags used (i.e. anti-*E*. *caproni*-actin, anti-*E*. *caproni*-enolase and anti-mouse IgA/G) was observed (Fig 1, S1 Movie, S2 and S3 Figs), indicating that the host immune response effectively targets *E*. *caproni* adults for antibody-mediated attack. Actin and enolase are immunogenic proteins, commonly found in ESPs of *E*. *caproni* and other trematodes, so that antibodies against both molecules were used as general markers to tag the parasite surface and the deposit of ESPs. Considering that distal tegument consists of a syncytial cytoplasm, externally limited by a plasma membrane, and that no detergents were employed for immunofluorescence, the ESP molecules detected on the parasite surface are outside the tegument itself. These results are in agreement with those from Simonsen and co-authors [3, 19], indicating that mouse antibodies bind to secreted antigens and form an external layer of immune complexes that covers the parasite. Moreover, Sotillo *et al*. [18] found that both molecules (actin and enolase) were among the most antigenic proteins in the ESPs of *E*. *caproni*.

The decrease in the fluorescent signal for *in vivo* bound antibodies on worm surface during *in vitro* incubation is shown in S4 Fig. Loss of surface-bound antibodies during *in vitro* culture has been previously described in *E*. *caproni* and other trematodes [1,3]. It was suggested that the shedding of surface antigens and, consequently, the antigen-bound antibodies, might be an adaptation of this group of parasites to withstand the host immune attack [20]. However, the approach we have followed herein, based on monitoring *in vitro* the dynamics of bound antibodies by double immunofluorescence, reveals a different mechanism of immune evasion. This new mechanism consists in entrapping the surface-bound antibodies within newly secreted products. Fig 1, S1 Movie and S3 Fig show how, as the time culture increases, an external layer of ESPs, stained in red (anti-*E*. *caproni* actin), appears over the *in vivo*-bound antibodies (seen in green/yellow). This new layer is almost continuous after 30 min of incubation in RPMI. Andresen *et al*. [20] found that antigen-antibody complexes were released from the parasite surface into the culture medium during no longer than 20–25 min, suggesting that surface turnover was completed by this time. Our results, however, yield a novel interpretation of this finding. It seems that the loss of surface-bound antibodies *in vitro* is not due to the turnover of surface antigens, but instead to the trapping of the antibodies underneath a layer of excreted/secreted molecules. Results were displayed for anti-IgA (Fig 1, S1 Movie, S2 and S3 Figs).

**Fig 2:**
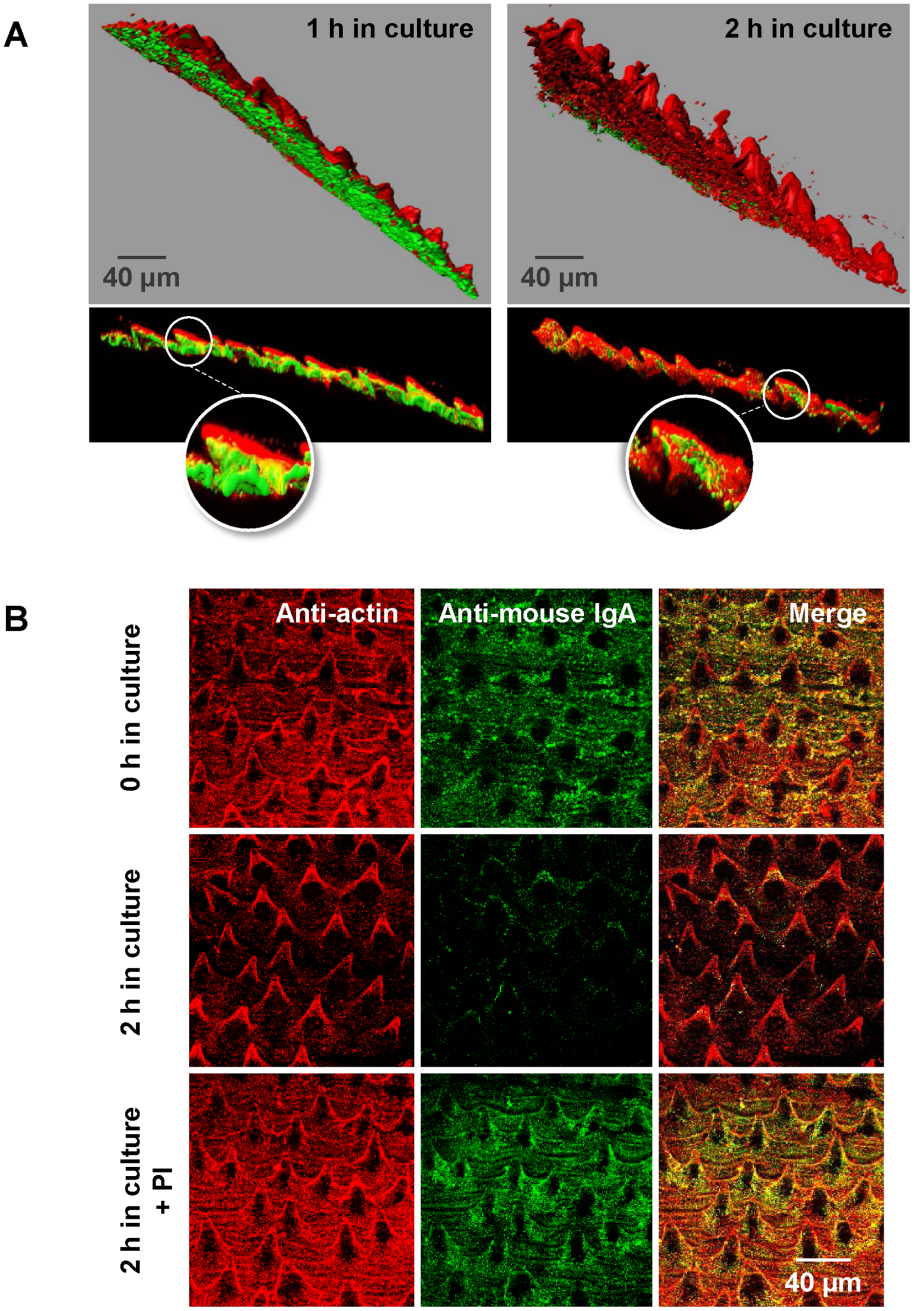
Degradation of trapped antibodies by parasite-derived proteases. (A) 3D surface modelling of the staining of *Echinostoma caproni* adults cultured *in vitro* for 1 and 2 h, respectively. Note that the green dye (*in vivo*-bound antibodies), which is under the red-dye layer (anti-*E*. *caproni* actin), has almost disappeared after 2 h in culture medium. (B) Z projection of the stained surface of *E*. *caproni* adults at 0 and 2 h of incubation in the presence and absence of protease inhibitors (PI).

A 3D reconstruction of antibody trapping and degradation process was created at two time points of *in vitro* culture using Imaris software (Fig 2A). After 1 h incubation, it can be appreciated how the layer of *in vivo*-bound antibodies is beneath a continuous and relatively thick layer of secreted material, tagged either with anti-actin or anti-enolase antibody. This indicates that antibodies are not lost from the surface of the parasite, as previously suggested [3, 20] but, in contrast, they become hidden beneath a layer of ESPs. After 2 h in RPMI, *in vivo*-bound antibodies are scarcely detected on the parasite surface, suggesting that trapped antigen-antibody complexes were removed or degraded somehow. Indeed, antibody degradation by parasite-secreted proteases is well recognized as a mechanism to evade the host immune response [21].

The *in vitro* study has let us elucidate the dynamics of bound-antibodies through the incubation of worms in an antibody-free medium. However, antibody trapping is expected to function also within the host. In that case, both antibody binding and trapping must occur simultaneously, and the continuous entrapment of surface-bound antibodies may serve to disable their harmful impact over the parasite. To confirm our hypothesis that this is a dynamic process and to examine the role that secreted proteases may have in the context of this mechanism of immune evasion, two different experiments were carried out.

Firstly, adult worms were incubated for 2 h in culture media in the presence and absence of a cocktail of protease inhibitors. As it was expected, only a slight reduction in the anti-IgA staining was observed after 2 h when protease inhibitors were added to the culture media (Fig 2B and S5 Fig). This result indicates that antibody trapping not only hampers the accessibility of other immune molecules and cells to the bound antibodies, but also facilitates antibody degradation by parasite-derived proteases. Furthermore, this proves that the loss of green staining (*in vivo*-bound mouse antibodies) *in vitro* is not an artifactual result. The fact that *in vivo*-bound antibodies are still detected over the surface after culture in the presence of protease inhibitors demonstrates that the reduction in anti-mouse antibody staining (green) during incubation in not-supplemented medium is not consequence of a passive release of antibodies due to low-affinity bindings (Fig 2B and S5 Fig). A variety of proteases have been previously detected in the ESPs of *E*. *caproni* [16, 22] and, using protease inhibitors, herein we have also shown that ESPs of *E*. *caproni* have protease activity. Future studies will show which proteases are involved in the degradation of entrapped antibodies on the parasite surface.

**Fig 3:**
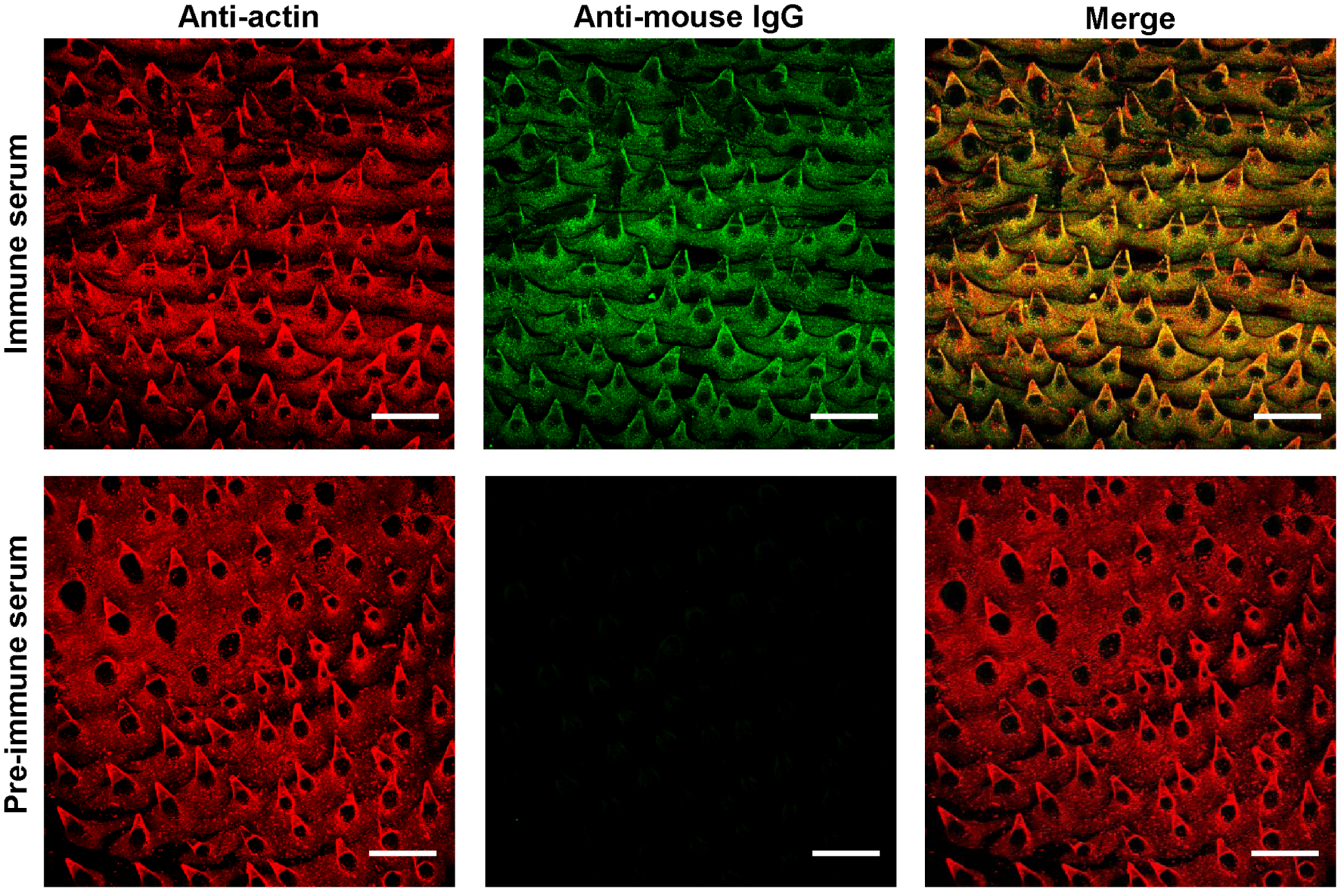
Binding of serum antibodies to *Echinostoma caproni* surface. Binding of specific, mouse serum antibodies (green) to the external layer of secreted products (red) that is formed over the surface of *E*. *caproni* adults during *in vitro* incubation. Scale bar = 50 μm.

Secondarily, to verify that the ESPs deposited over the surface of the parasite can be recognized by new antibodies, adult specimens were incubated for 1 h with the serum of *E*. *caproni*-infected mice and a HRP-conjugated anti-mouse IgG as a secondary antibody. Previously, these worms had been kept for more than 2 h in RPMI to ensure the elimination of *in vivo*-bound antibodies. Immune mouse sera effectively tagged the surface of worms, whereas unspecific antibody binding was not observed over those incubated with pre-immune serum, indicating that ESPs accumulated on the surface can be the target for new antibodies (Fig 3). Furthermore, this confirms that antibody binding to parasite antigens is specific and that nonspecific unions, e.g. through Fc, do not occur. Altogether, these findings suggest a constant and reciprocal interplay between parasite- and host-released molecules at the site of infection. Antibody responses have been proven to have little effect on worm survival and development in *E*. *caproni* primary infections, with the highest rates of establishment and longevity being associated with high levels of mucosal antibodies [12, 13]. The evasion mechanism described herein may serve to explain the lack of effectiveness of these responses.

**Fig 4:**
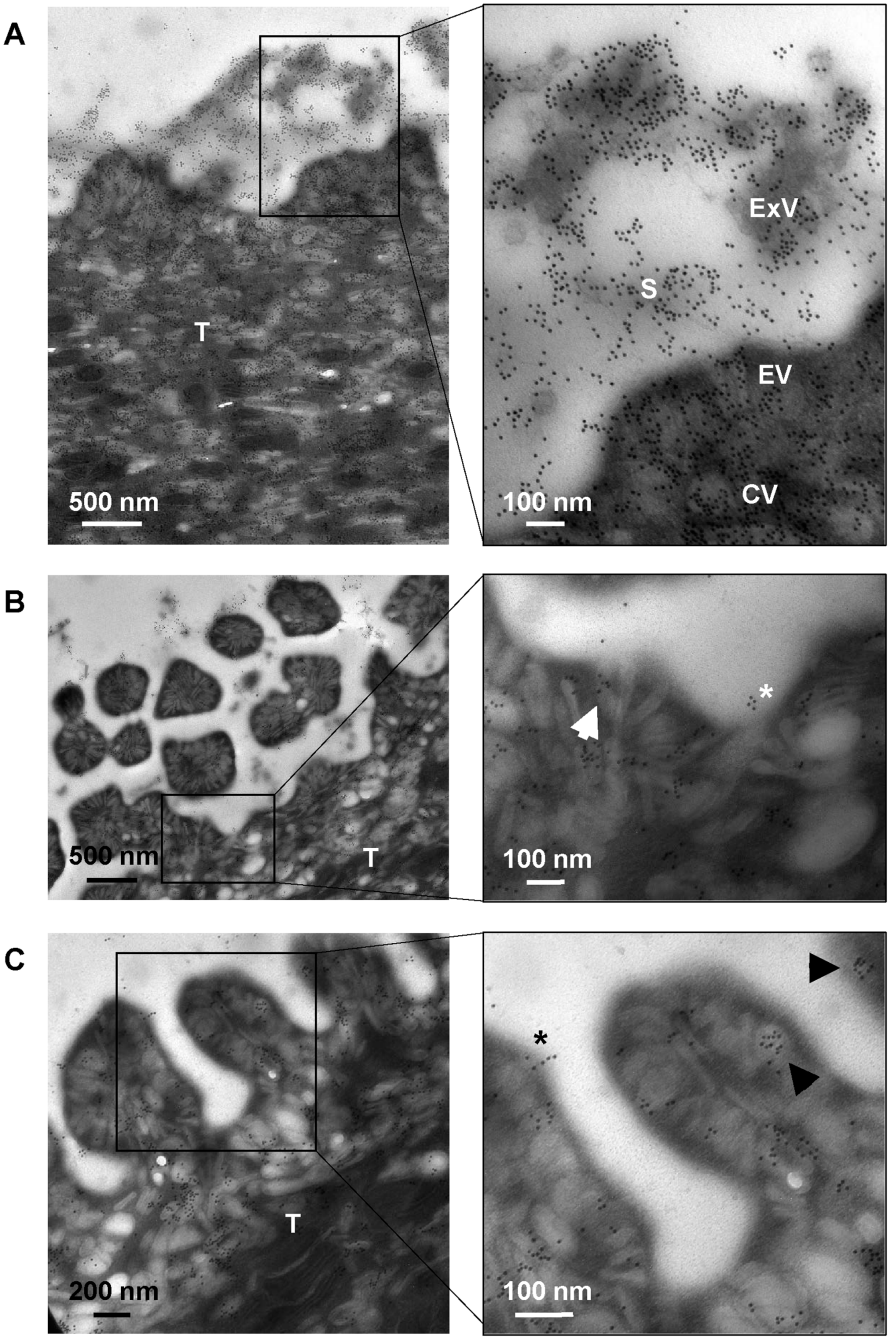
Immunogold labelling of *Echinostoma caproni* tegument. Transmission electron micrographs of the tegument stained with rabbit sera against *E*. *caproni* excretory/secretory products (A) and *E*. *caproni* actin (B and C). (B) arrow pointed to an elongated vesicle containing actin molecules, which is opened in the tegument apex. Asterisk indicates secreted actin molecules in the vicinity of an opened secreting vesicle. (C) anti-actin staining inside circular vesicles (arrows) and over the surface, in the vicinity of an opened vesicle (asterisk). T: tegument; ExV: extracellular vesicle; S: secretions; EV: elongated vesicles: CV: circular vesicles.

**Fig 5:**
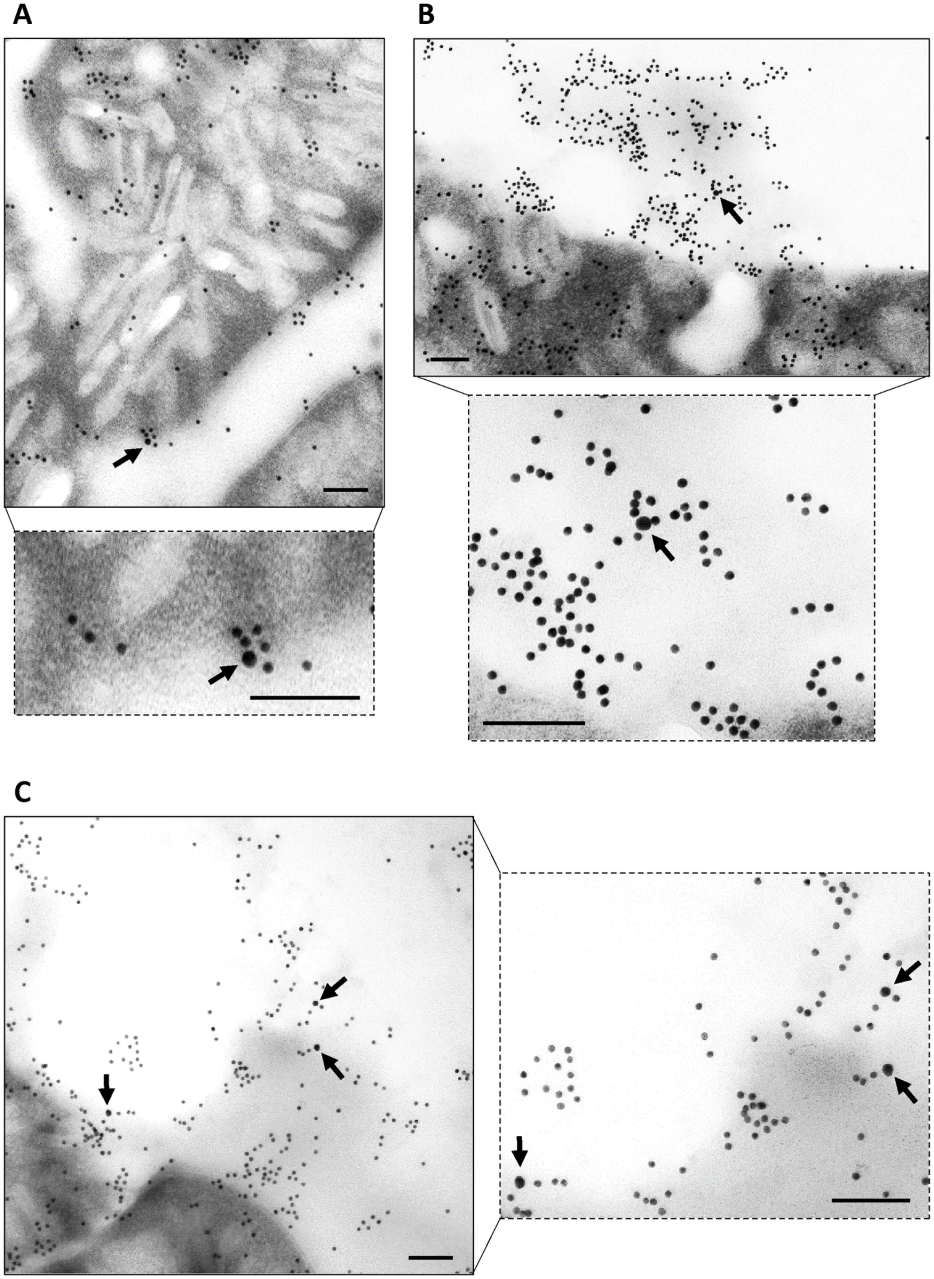
Confirmation of antibody trapping by double immunogold. Transmission electron micrographs of the *Echinostoma caproni* tegument, double stained with rabbit sera against *E*. *caproni* excretory/secretory products (detected by a secondary antibody coupled to 12 nm colloidal gold) and an anti-mouse IgG conjugated with 18 nm colloidal gold. Images show antibody trapping by secreted products on the worm surface (A) and within the extracellular secretions in the tegument proximity (B and C). Arrows point to 18 nm colloidal gold particles. Scale bars = 100 nm.

**Fig 6:**
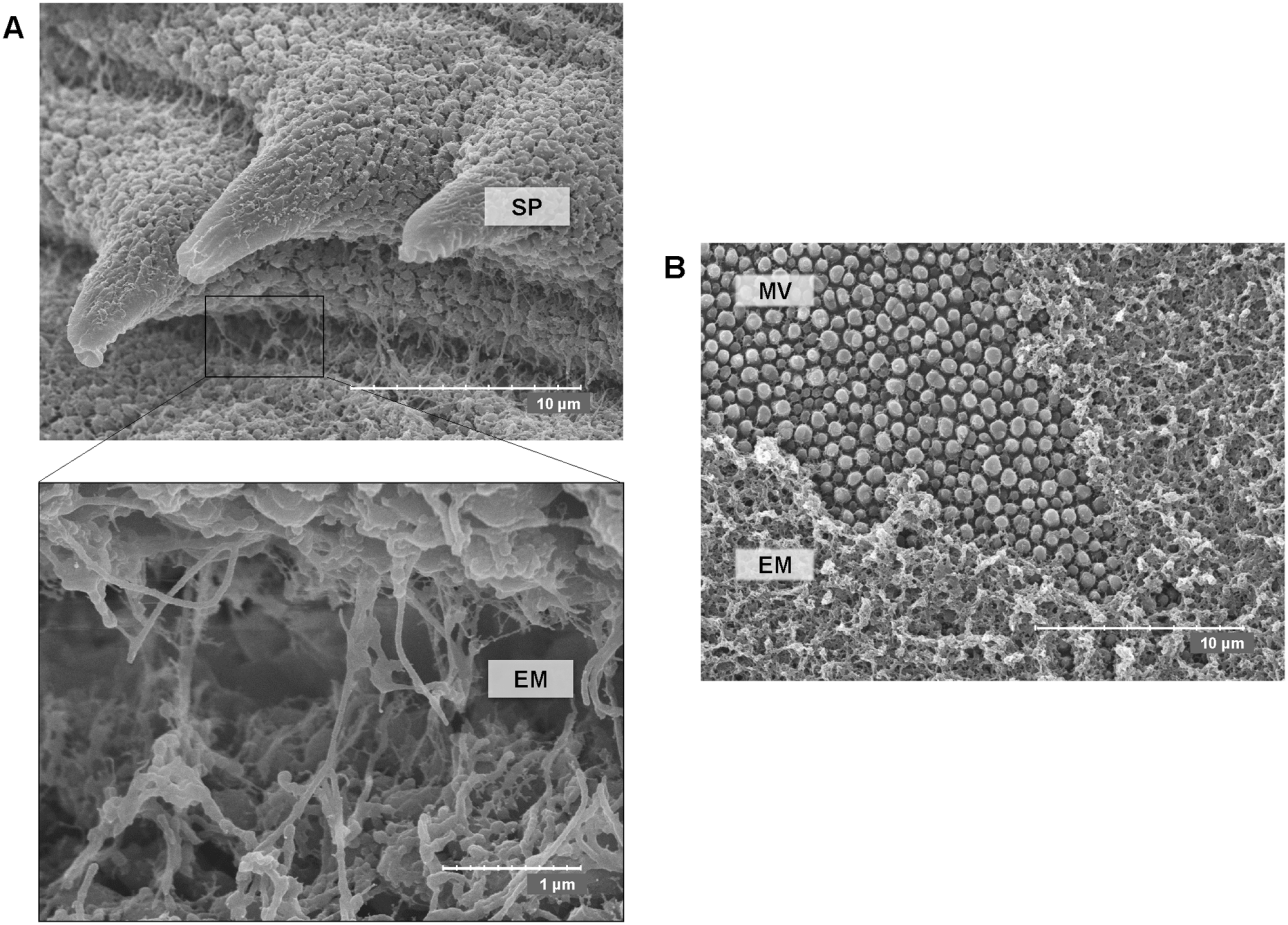
Scanning electron microscopy (SEM) of ventral and dorsal *Echinostoma caproni* surfaces. SEM micrographs showing extracellular material (EM) adhered to the outer tegumental membrane. SP: spine; MV: microvilli. (A) ventral side and (B) dorsal side. The dots in the scale bars delimitate 1/10 of the length indicated in the figure.

To examine if the proposed mechanism is compatible with the tegument dynamics and ultrastructure, the surface of *E*. *caproni* was studied by transmission and scanning electron microscopy (TEM and SEM, respectively). Moreover, immunogold labelling using anti-actin and anti-ESPs polyclonal antibodies was performed. Secretory vesicles of different morphology are highly abundant in the tegumental syncytium, indicating a very active secreting surface. Elongate and circular vesicles [20] accumulate manly at the apex, where they fuse with the external plasma membrane and empty their content to the outside (Fig 4 and S6 Fig). Immunogold labelling with anti-*E*. *caproni* ESPs showed a widespread staining of both the tegumental syncytium and the parasite surroundings, i.e. external surface, secretions and extracellular vesicles (Fig 4A). Gold particles inside membrane-bound vesicles, either elongated or circular, are seen profusely thorough the syncytium (Fig 4A). Similar results were observed with anti-*E*. *caproni* actin, though specific staining was much less extensive as could be expected when detecting a discrete molecule (Fig 4B and 4C). Unstained negative control is shown in S6 Fig. This demonstrates that secreted proteins are incorporated in tegumentary vesicles that fuse with the plasma membrane in the apex and discharge their content to the extracellular milieu. In Fig 4B, specific anti-actin staining is seen inside an elongated vesicle opened in the apex and in the vicinity of an opened vesicle. Packing of actin molecules within apical circular vesicles is shown in Fig 4C. According to these results, antibody trapping by newly secreted products is mechanistically feasible and it may occur *in vivo*. This was further confirmed by double immunogold for ESPs and mouse antibodies, showing that host antibodies are trapped by ESPs both on the parasite surface (Fig 5A) and within the extracellular secretions in the tegument vicinity (Fig 5B and 5C). High-resolution SEM reveals that a layer of extracellular material is deposited on the parasite surface, both on the ventral and dorsal sides (Fig 6). Highly likely, this layer consists of a mixture of parasite secreted proteins and host-derived molecules (i.e. trapped antibodies) and corresponds to what is detected by double indirect immunofluorescence.

Although we cannot discard that antibody shedding effectively operates to evade the host immune response, our results indicate that it is less relevant than the mechanism proposed herein. Antibody shedding was suggested as a mechanism of immune evasion based on the facts that adult worms lost surface-bound antibodies during *in* vitro culture [3] and they rapidly released antigen-antibody complexes into the culture medium [20]. Hence, it was hypothesized that bound antibodies were removed from the surface due to antigen turnover. Our results of confocal microscopy show that, in culture, most of antibodies are not shed, as suggested by Andresen *et al*. [20], but they remain bound to the surface beneath a layer of ESPs. Loss of antibodies from the parasite surface is mainly related to an initial trapping by ESPs, which masks surface-bound antibodies, and the subsequent degradation of trapped antibodies by parasite-derived proteases. The fact that surface-bound antibodies became entrapped within a layer of ESPs may explain the interpretation of Andresen and co-workers [20], since this layer prevents antigen-antibody shedding, thus antibodies were non-detectable by the modified ELISA method used by those authors. The question that raises, however, is how newly secreted antigens get to cover the layer of surface-bound antibodies. In view of our results, it is tempting to hypothesize that discrete antigens are able to diffuse through the layer of antigen-antibody complexes, generating a gradient of ESPs from the parasite to the outside that gets to cover the antibody layer. As secreted antigens are not anchored, the most external molecules are progressively released into the medium, thus the covering of antibodies is maintained due to the continuous secretion of ESPs. In contrast to discrete molecules, antigen-antibody complexes are not expected to diffuse due to their greater size, becoming entrapped within this mesh of antigens, which further facilitates their retention.

*In vivo*, antibody binding and trapping by ESPs is a continuous process in which antibodies and ESPs are overlapping. In this context, parasite-derived proteases may play a critical role by degrading the layers of molecules that are continuously formed on the parasite surface. Despite the complexity of this process, our experimental design has allowed to determine how it occurs and its potential consequences on parasite survival within a hostile environment.

In conclusion, the results presented in this paper lead to a new interpretation of classic studies on tegument dynamics in parasitic trematodes. As with other common evasion mechanisms (i.e. antigen shedding or protease cleavage), antibody trapping and degradation is expected to function in other helminth parasites in addition to *E*. *caproni*, alleviating the deleterious effects of antibodies and promoting parasite survival. Our current results suggest that antibody trapping may occur through the covering of surface-bound antibodies with secreted antigens. Nevertheless, future studies with *E*. *caproni* and other helminths are needed to elucidate how the trapping process happens *in vivo* and which proteases are specifically involved in antibody degradation. Helminth infections affect millions of people worldwide, mainly in the poorest regions, and have a tremendous economic impact in the livestock sector. In the context of host-parasite relationships, the immune evasion mechanism described herein may help to understand the limited effectiveness that the antibody responses, *per se*, have against this group of parasites.

## Supporting information

S1 Fig Confirmation of antibody specificity. The specificity of the antibodies employed for double indirect immunofluorescence and/or immunogold labeling was tested by western blot. Excretory/secretory products (ESPs) were electrophoresed, blotted on nitrocellulose membrane and incubated against anti-*Echinostoma caproni* (Ec)-actin (lane 1), anti-Ec-enolase (lane 2) and anti-Ec ESPs (lane 3), all three performed in rabbit. Pre-immune rabbit serum was used as negative control (lane 4).
(TIF)

S2 Fig Targeting of *Echinostoma caproni* surface with anti-enolase antibody at increasing time of *in vitro* incubation. 2D-images from laser confocal microscopy on the surface of *E*. *caproni* adults stained with anti-*E*. *caproni* enolase (red) and anti-mouse IgA (green). Merge is shown in yellow. For each time point, representative images of XY and YZ axes are shown (left and right, respectively). Zero minutes incubation refers to worms that were fixed immediately after being removed from the intestine of the mouse.
(TIF)

S3 Fig Double immunofluorescent staining of *Echinostoma caproni* surface at increasing time of *in vitro* incubation. 2D-images from laser confocal microscopy on the surface of *E*. *caproni* adults stained with anti-*E*. *caproni* actin (red) and anti-mouse IgG (green). Merge is shown in yellow. For each time point, representative images of XY and YZ axes are shown (left and right, respectively). Zero minutes incubation refers to worms that were fixed immediately after being removed from the intestine of the mouse.
(TIF)

S4 Fig Quantitation of anti-IgA staining. Loss of anti-IgA staining on worm surface along time is shown as the decrease in the percentage of image area covered by anti-HRP, FITC-conjugated, antibody (% AC). Vertical bars show standard deviation and asterisks indicate statistical differences for each incubation time in relation to non-incubated worms (0 min) (*p*<0.0001).
(TIF)

S5 Fig Quantitation of anti-IgA staining after incubation with protease inhibitors. Anti-IgA staining on worm surface was calculated as the percentage of image area covered by anti-HRP, FITC-conjugated, antibody (% AC) in worms incubated for 120 min in the presence and absence of protease inhibitors (PI). Vertical bars show standard deviation and asterisks indicate statistical differences in relation to non-incubated worms (0 min) (*p*<0.0001).
(TIF)

S6 Fig Transmission electron micrograph of a negative control for immunogold labelling. Negative controls were processed as samples but incubating the grids with pre-immune rabbit sera as primary antibody. T: tegument; ExV: extracellular vesicle; S: secretions.
(TIF)

S1 Movie Laser confocal microscopy on the surface of *Echinostoma caproni* adults. Double indirect immunofluorescence with anti-actin (red) and anti-mouse IgA (green). Merge is shown in yellow. For each time point, staining through the Z-axis is shown. Zero minutes incubation refers to worms that were fixed immediately after being removed from the intestine of the mouse.
(MP4)
