# Supplementary figures and images for: Antibody trapping: A novel mechanism of parasite immune evasion by the trematode Echinostoma caproni
Source: PLoS Negl Trop Dis. 2017 Jul 17;11(7):e0005773. doi: 10.1371/journal.pntd.0005773 (PMC5531663; doi:10.1371/journal.pntd.0005773)

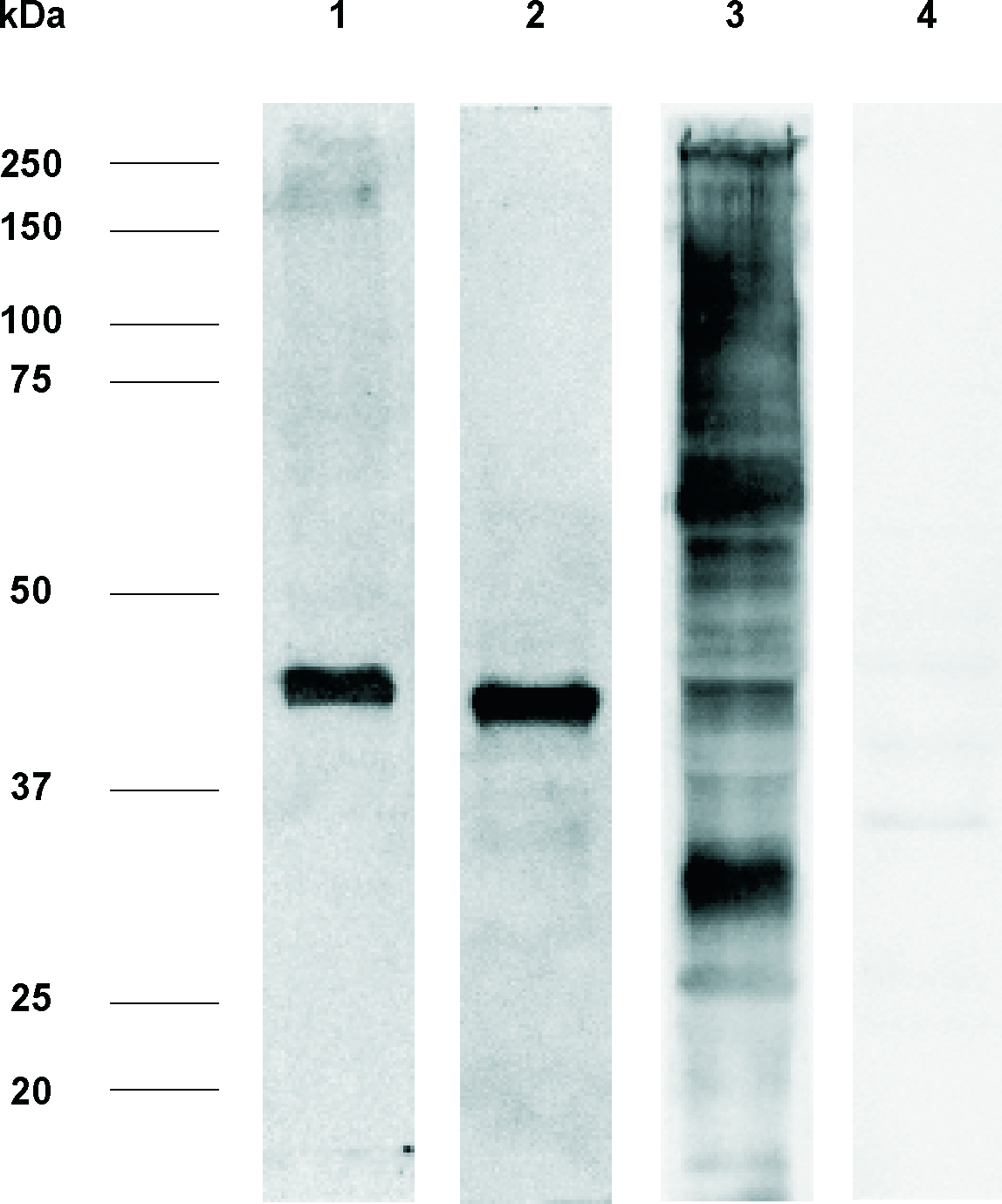

Supplement: S1 Fig — The specificity of the antibodies employed for double indirect immunofluorescence and/or immunogold labeling was tested by western blot. Excretory/secretory products (ESPs) were electrophoresed, blotted on nitrocellulose membrane and incubated against anti-Echinostoma caproni (Ec)-actin (lane 1), anti-Ec-enolase (lane 2) and anti-Ec ESPs (lane 3), all three performed in rabbit. Pre-immune rabbit serum was used as negative control (lane 4). (TIF) [file pntd.0005773.s001.tif]

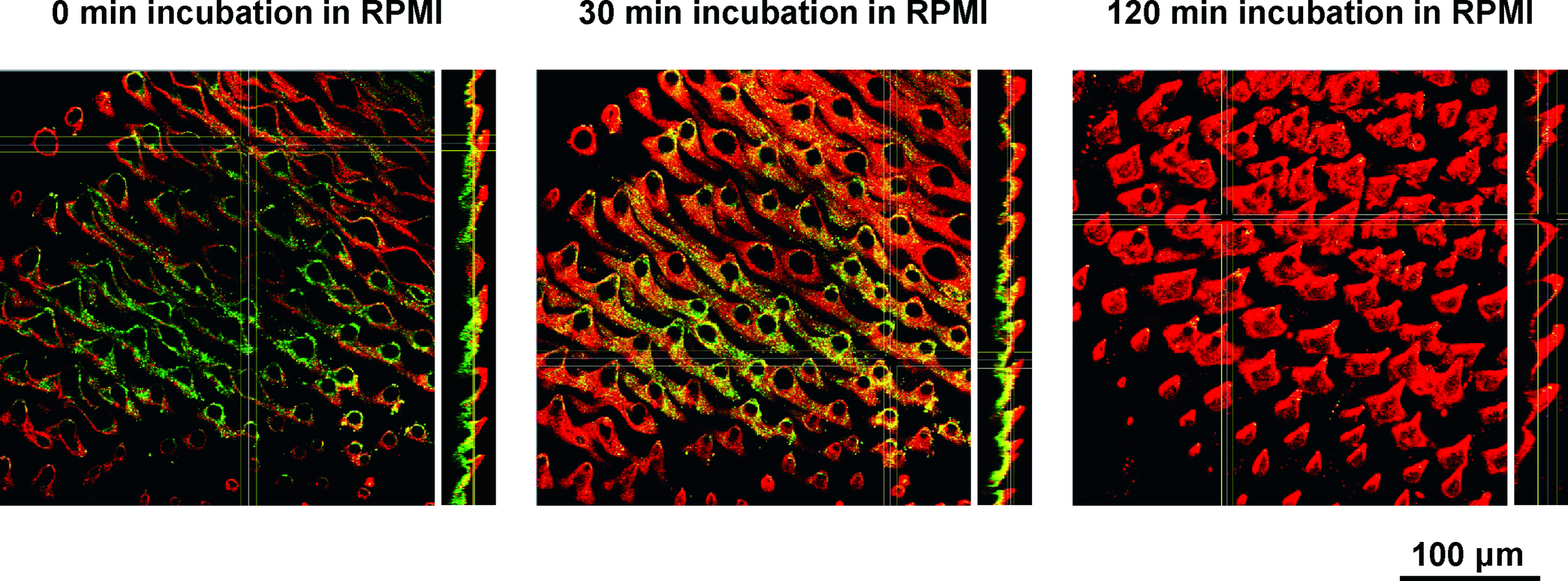

Supplement: S2 Fig — 2D-images from laser confocal microscopy on the surface of E. caproni adults stained with anti-E. caproni enolase (red) and anti-mouse IgA (green). Merge is shown in yellow. For each time point, representative images of XY and YZ axes are shown (left and right, respectively). Zero minutes incubation refers to worms that were fixed immediately after being removed from the intestine of the mouse. (TIF) [file pntd.0005773.s002.tif]

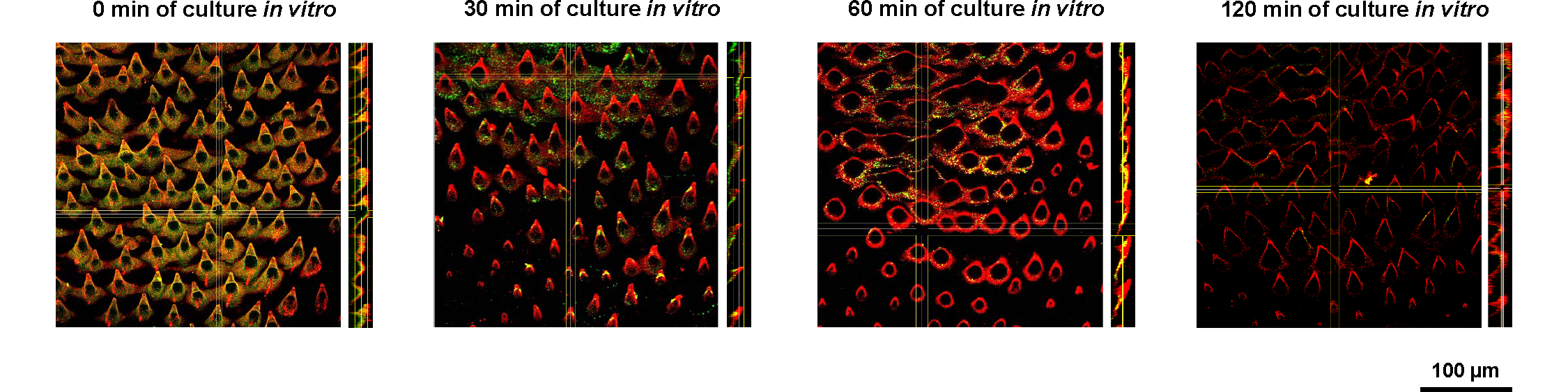

Supplement: S3 Fig — 2D-images from laser confocal microscopy on the surface of E. caproni adults stained with anti-E. caproni actin (red) and anti-mouse IgG (green). Merge is shown in yellow. For each time point, representative images of XY and YZ axes are shown (left and right, respectively). Zero minutes incubation refers to worms that were fixed immediately after being removed from the intestine of the mouse. (TIF) [file pntd.0005773.s003.tif]

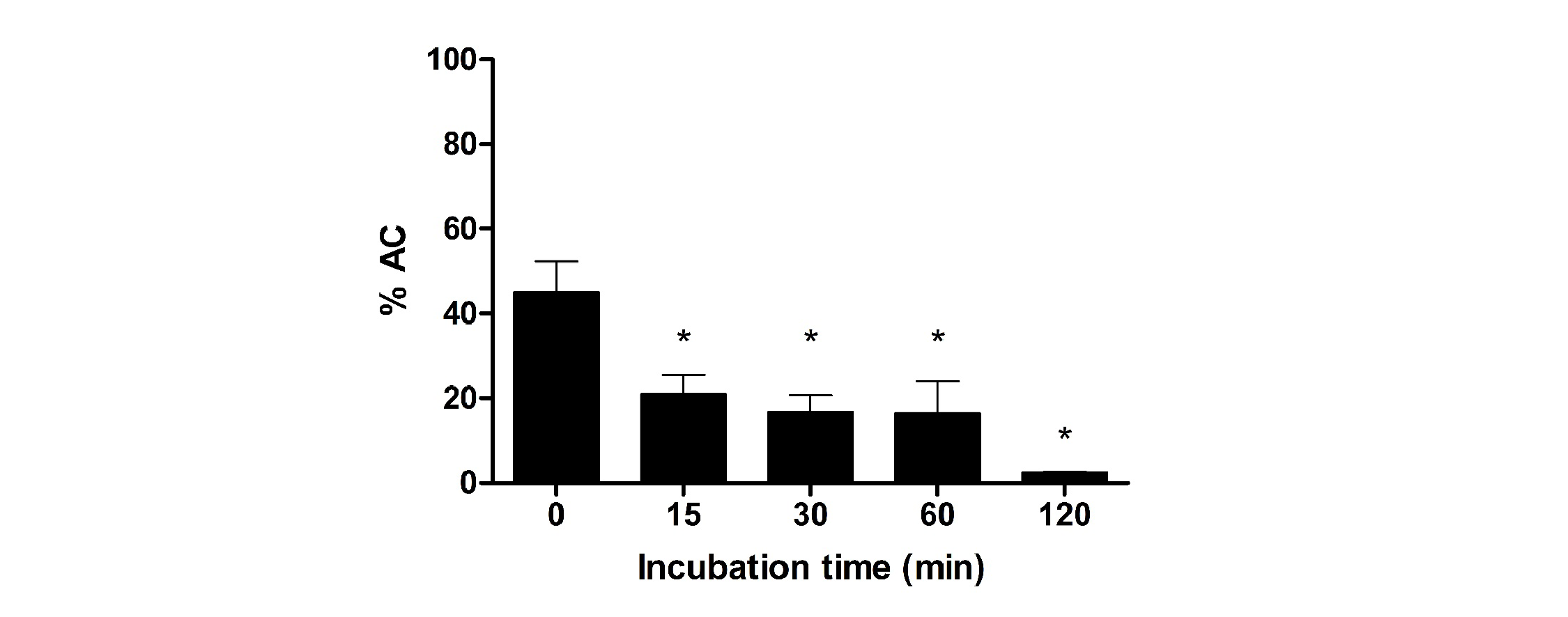

Supplement: S4 Fig — Loss of anti-IgA staining on worm surface along time is shown as the decrease in the percentage of image area covered by anti-HRP, FITC-conjugated, antibody (% AC). Vertical bars show standard deviation and asterisks indicate statistical differences for each incubation time in relation to non-incubated worms (0 min) (p<0.0001). (TIF) [file pntd.0005773.s004.tif]

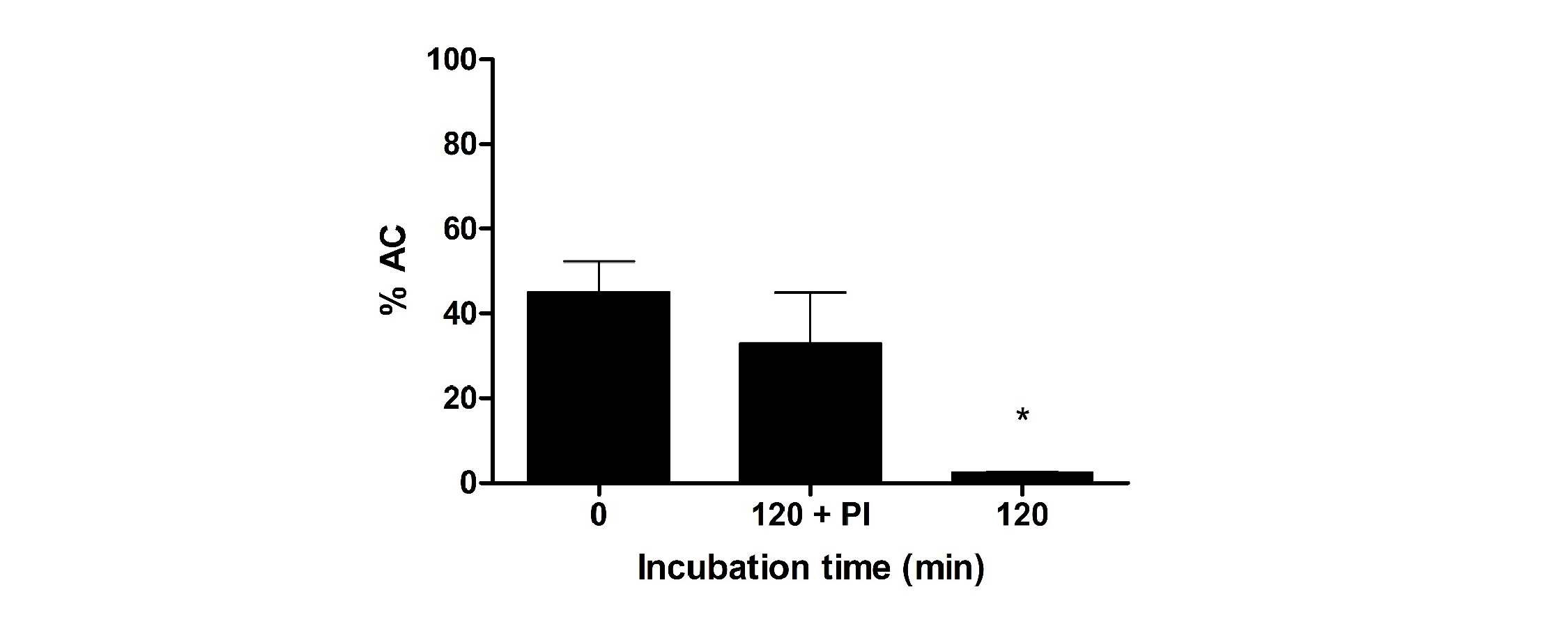

Supplement: S5 Fig — Anti-IgA staining on worm surface was calculated as the percentage of image area covered by anti-HRP, FITC-conjugated, antibody (% AC) in worms incubated for 120 min in the presence and absence of protease inhibitors (PI). Vertical bars show standard deviation and asterisks indicate statistical differences in relation to non-incubated worms (0 min) (p<0.0001). (TIF) [file pntd.0005773.s005.tif]

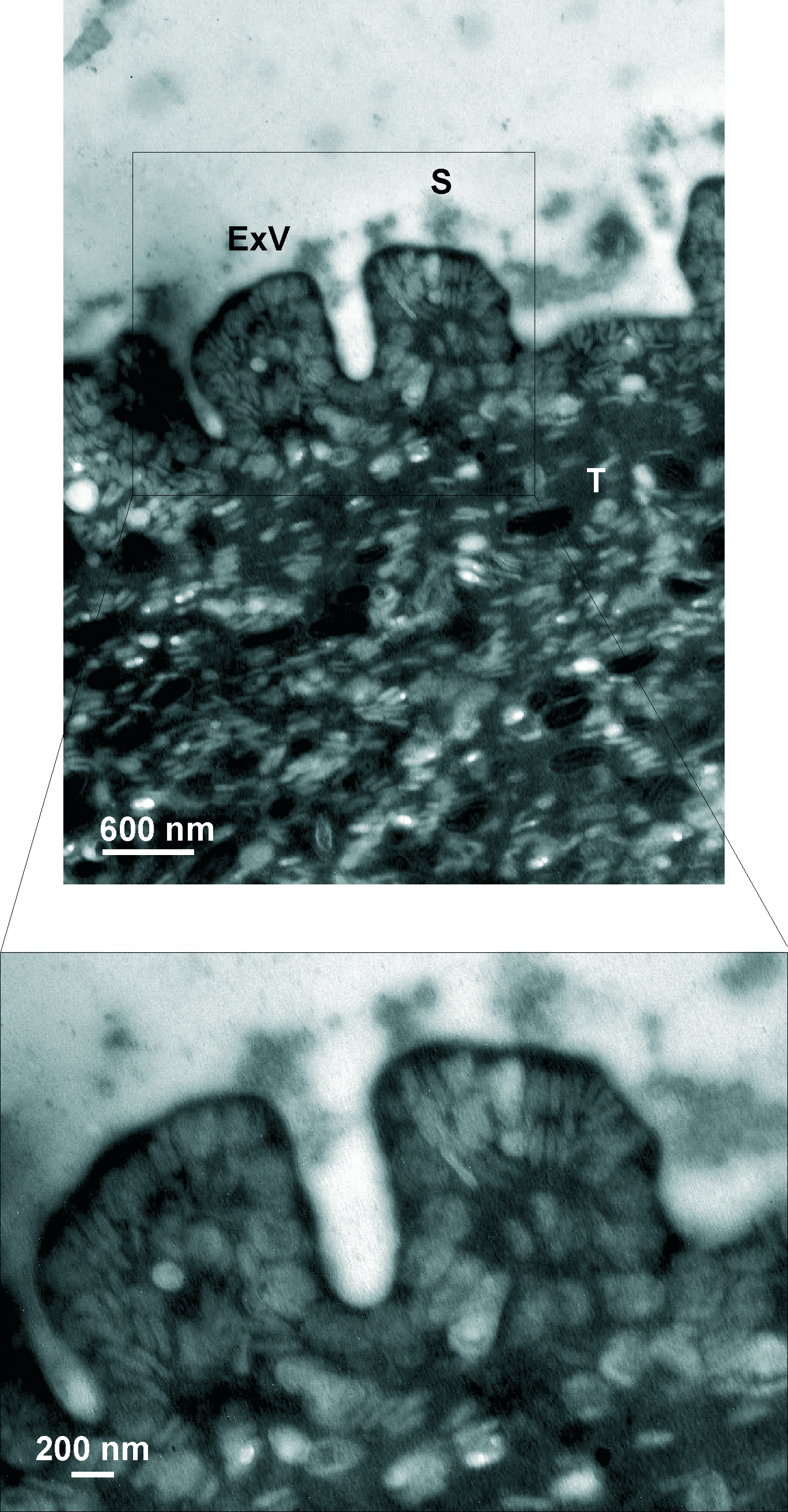

Supplement: S6 Fig — Negative controls were processed as samples but incubating the grids with pre-immune rabbit sera as primary antibody. T: tegument; ExV: extracellular vesicle; S: secretions. (TIF) [file pntd.0005773.s006.tif]
